# Effect of Polydopamine/Sodium Dodecyl Sulfate Modified Halloysite on the Microstructure and Permeability of a Polyamide Forward Osmosis Membrane

**DOI:** 10.3390/membranes13070638

**Published:** 2023-06-30

**Authors:** Jie Yu, Weiqi Jing, Eryong Liu, Shuangming Du, Hui Cai, Huiling Du, Jinlei Wang

**Affiliations:** School of Materials Science and Engineering, Xi’an University of Science and Technology, Xi’an 710054, China; 17391934747@163.com (J.Y.); 18392606130@163.com (W.J.); smdu@xust.edu.cn (S.D.); caihui35806505@163.com (H.C.); hldu@foxmail.com (H.D.);

**Keywords:** emergency rescue, forward osmosis membrane, compound modification, microstructure, permeability

## Abstract

Mine water cannot be directly consumed by trapped people when a mine collapses, so it is difficult for people to carry out emergency rescues to ensure their safety. Therefore, a water bag made of a forward osmosis (FO) membrane has been designed that can efficiently filter coal mine water to meet the urgent needs of emergency rescue. Before interfacial polymerization (IP), sodium-dodecyl-sulfate-modified halloysite (SDS−HNT) was added to an MPD aqueous solution to prepare an SDS−HNT polyamide active layer, and then the prepared membrane was placed into a polydopamine (PDA) solution formed by the self-polymerization of dopamine and a PDA/SDS−HNT composite film was prepared. The results showed that the original ridge−valley structure of the polyamide membrane was transformed to a rod-, circular-, and blade-like structure by the addition of SDS−HNTs. Subsequently, a dense PDA nanoparticle layer was formed on the modified membrane. The polyamide/polysulfone forward osmosis membrane modified by co-doping of PDA and SDS−HNTs displayed both the best water flux and rejection rate, confirming the synergistic effect of compound modification. Therefore, the high-performance permeability of the polyamide membrane modified by SDS−HNTs and PDA provides great convenience for the emergency filtration of coal mine water, and also has potential applications in wastewater treatment and seawater desalination.

## 1. Introduction

With the increasing depth of coal mining, the emergency rescue of coal mine accidents has become more difficult. In particular, essential drinking water will greatly protect trapped people. After the process of coal mining, the ubiquitous mine water is impossible to drink directly, owing to the acid pollution, suspended matter, turbidity, hardness, sulfates and fluorides. A previous study of coal mines in northern Shaanxi showed that the main pollutants of mine water were sulfate and fluoride, which were 3 times higher for sulfate and 5 times higher for fluoride than the drinking water standard (GB/T 5749−2006) [1]. Therefore, emergency technology that can filter mine water into drinking water will effectively extend the lives of trapped people. Weiqi Jing et al. assembled a forward osmosis membrane in a portable water bag, and the water bag produced was successfully applied in an emergency rescue by Shendong Coal Mine Company (Yulin, China) [2].

As a new water treatment technology, membrane technology has been widely used in wastewater treatment. Common membrane filtration technologies, such as reverse osmosis (RO), nanofiltration (NF), microfiltration (MF), ultrafiltration (UF) and forward osmosis (FO), can effectively filter harmful ions and organic substances in wastewater [3,4,5]. Among them, FO is a new membrane separation technology powered by the chemical potential or osmotic pressure difference between the feed and discharge solutions, which has showed potential in emergency filtration of mine water [6,7]. In addition, forward osmosis technology has also shown good application prospects in the fields of sewage treatment, power generation, food and medicine preparation, aerospace, military emergencies and so on [8,9,10].

Among various forward osmosis membranes, a polyamide active layer and a polysulfone supporting layer are considered as the most effective materials to prepare two-layer FO membrane structures. The ideal permeability can be obtained by the modification of the active layer and supporting layer, respectively. Researchers have optimized film nanocomposites by adding hydrophilic nanomaterials to the support layer or by adding monomer solutions (such as an MPD aqueous solution or a TMC/hexane solution) to the active layer [11,12,13]. The hydrophilicity of the support layer can be improved by selecting materials with good hydrophilicity and by surface modification of the support layer [14]. For4 example, polyethersulfone (PES) was modified by polydopamine (PDA), and the polyamide active layer was modified by adding piperazine (PIP) and trimethyl chloride (TMC) to the MPD aqueous solution. The results showed [15] that the PES support layer rendered by active groups not only enhanced the interfacial strength between the support layer and the active layer, but also improved the water flux. Among them, the polysulfone supporting layer of the FO membrane composed of sponge-like and finger-like structures provided good strength to bear a higher hydraulic pressure [16]. The polyamide active layer showed great application prospects in forward osmosis fields, owing to its excellent select transmission performance, good flexibility and low cost. In contrast to the supporting layer, the selective permeability, stain resistance, antibacterial properties and self-cleaning properties of the active layer can easily be improved by the addition of different materials [17,18]. In particular, improving the selectivity of the polyamide layer is also the main modification method for polyamide/polysulfone forward osmosis membranes. In order to achieve excellent permeability, many researchers have successively modified the polyamide active layer by adding nanoparticles such as zeolite [19], carbon nanotubes (CNTs) [20], silica [21], molybdenum disulfide (MoS_2_) [22] and titanium dioxide (TiO_2_) [23]. However, the water flux of the existing modified polyamide/polysulfone forward osmosis membranes is about 10–30 Lm^−2^h^−1^, restricting the water treatment efficiency in emergency rescues [24,25,26,27].

Halloysite nanotubes (HNTs) are two-dimensional hollow nanomaterials with good hydrophilicity, large aspect ratios and a certain pollution resistance [28], so they are frequently used as active layer modifiers for the preparation of TFC membranes. Shah et al. used polydopamine (PDA) and halloysite (HNTs) as interlayer modifiers to prepare highly hydrophilic nanocomposite (TFC) films [29]. The test results showed that HNTs had a promoting effect on the formation of a highly cross-linked thick active layer. In addition, the water flux of HNT composite membranes increased to 26.9 Lm^−2^h^−1^, which may be related to the abundance of hydrophilic hydroxyl groups in HNTs. The study of Tzounis et al. [30] showed that HNTs embedded in a PSF substrate are an attractive option to reduce the thickness of the polyamide membrane, for example, the thicknesses of the PSF substrate were 0.95 mm and 0.37 mm for a polyamide membrane with 0.5 wt. % hydrophilic HNTs. Furthermore, the water flux of composite membranes with HNTs increased two-fold, which could be attributed to the abundant hydrophilic hydroxyl groups in HNTs. Ghanbari et al. demonstrated that the roughness, hydrophilicity and anti-fouling of polyamide membranes were enhanced by the addition of TiO_2_/HNT nanomaterials [31,32]. However, due to the agglomeration of HNTs, improving the dispersion of HNTs in the polyamide membrane was the key to a successful modification. Accordingly, the uniform dispersion of HNTs was also the key to ensure a high water flux and an excellent retention rate of the forward osmosis membrane [33].

Polydopamine (PDA), often used for hydrophilic surface modification, is a kind of polymer with oxygen-containing functional groups (−OH) [34]. It has attracted widespread attention as a surface modifier for coatings due to its surface adhesion and anti-fouling properties. Previous studies have indicated that polyamide/polysulfone forward osmosis membranes with PDA possess excellent hydrophilicity, and the thickness of the PDA layer plays a dominant role in permeability [2,35]. Furthermore, the studies of Kallem et al. suggested that the hydrophilicity and water flux significantly improved by co-doping with PDA and TiO_2_, and this is beneficial for use in the recovery of oily wastewater [36]. Hao Guo et al. revealed the significant anti-fouling performance of PDA in FO membranes, providing new ideas for the cyclic service life of water bags [37]. Therefore, appropriate modification is also an important method to improve the permeability of polyamide/polysulfone forward osmosis membranes [38].

Based on this, in order to improve the permeability of polyamide/polysulfone forward osmosis membranes, halloysite nanotubes were selected to modify the polyamide layer. Meanwhile, a suitable method was used to improve the dispersion of halloysite nanotubes in the polyamide layer. Furthermore, hydrophilic surface modification also had an obvious effect on the hydrophilicity, permeability and selectivity of the forward osmosis membrane. Although this work has been extensively studied, the prepared composite FO membrane has been assembled into a portable water bag and equipped at the emergency rescue site of Shendong Coal Mine, providing frontline help for trapped people in the mine. A systematic study was conducted on the filtration and anti-fouling performance of composite membranes in practical environments. The related research had a guiding role in improving the permeability of polyamide forward osmosis membranes; in particular, a higher water flux is important for the application of forward osmosis membranes in emergency rescues.

## 2. Experimental Materials and Methods

### 2.1. Materials

Polyester screens (180 mesh) were purchased from Hangzhou Daheng Filter Cloth Co., Ltd. (Hangzhou, China). Polysulfone (PSF), N−methyl−2−pyrrolidone (NMP, AR), N, N−dimethylformamide (DMF, AR), m−phenylenediamine (MPD, AR), 1,3,5−benzenetricarbonyl trichloro (TMC, AR), dopamine hydrochloride (DA, AR), tris (hydroxymethyl) aminomethane (Tris, AR), sodium chloride (NaCl), lithium chloride (LiCl) and magnesium sulfate (MgSO_4_) were obtained from Sinopharm Chemical Reagent Co., Ltd. (Shanghai, China). Halloysite nanotubes (HNTs) were purchased from Sigma-Aldrich Co., Ltd. (St. Louis, MO, USA).

### 2.2. Fabrication of Polysulfone Membrane

The phase conversion method was used to prepare the polysulfone substrate membrane. First, 12 wt.% PSF was dissolved in NMP, followed by sequentially adding DMF and LiCl to the solution, where the mass ratio of DMF to NMP was 1:3. The prepared solution was stirred at 80 °C for 12 h, after which it was left to defoam for 24 h. Next, the cast solution was uniformly coated on the fixed polyester screen with a scraper with an air gap of 150 μm, and phase transformation was carried out at room temperature. After that, the phase transformation was completed before placing the prepared membrane bubbles in deionized water to remove the excess organic matter, and finally the polysulfone substrate membrane was obtained.

### 2.3. Fabrication of SDS−HNTs

An amount of 5 g of dried halloysite nanotubes and 0.61 g of sodium dodecyl sulfate (SDS) anionic surfactant were placed into 0.5 L of deionized water and mechanically stirred for 14 h [39]. After a suspension mixture was formed, it was centrifuged at 8000 rpm for 5 min, and then the supernatant was heated at 80 °C for another 24 h. Finally, it was ultrasonicated for 2 h for use [39].

### 2.4. Fabrication of the Polyamide/Polysulfone Composite Membrane

Polyamide membrane layers were prepared by the interfacial polymerization method. First, the PSF support layers were immersed in a 3.4 wt.% MPD aqueous solution for 2 min, stirred with magnetic heating and then excess MPD solution was removed from the surface using an air knife. Next, the membranes were immersed in 0.15 wt.% TMC hexane solution for 60 s to form an ultrathin polyamide membrane. Then, the composite membranes were cured in deionized water at 95 °C for 120 s. Finally, the excess organic matter on the membrane surface were rinsed and decomposed by a 200 ppm NaClO_3_ aqueous solution and a 1000 ppm NaHSO_3_ aqueous solution, and then heated and cured again to obtain a TFC membrane. The FO membrane was modified by adding 1 wt.% HNTs nanoparticles and 0.5 wt.%, 1 wt.%, 2 wt.% and 3 wt.% SDS−HNT nanoparticles to the MPD aqueous solution. During this process, SDS not only improved the uniform dispersion ability of HNTs, but also affected the IP process, causing chaos in the internal state of the TFC membrane and changing the micromorphology of the modified membrane [40]. The obtained samples were denoted as TFC−H1, TFC−S−H0.5, TFC−S−H1, TFC−SH−2, TFC−S−H3 and TFC−S−H1−PDA.

### 2.5. Fabrication of Dopamine Composite Forward Osmosis Membrane

A concentration of 0.01 mol/L tris was added to a 2 mol/L dopamine solution and the pH was adjusted to 8.5 with dilute hydrochloric acid. Next, the sample TFC−S−H1 membrane was removed and immersed in dopamine solution for 2 h, then rinsed thoroughly with deionized water and dried for use. The resulting sample was recorded as TFC−S−H1−PDA.

### 2.6. Characterization of Membranes

Field emission scanning electron microscopy (Hitachi S−4800, Hitachi, Ltd., Tokyo, Japan) and atomic force microscopy (SPM−9700HT, Shimatsu, Ltd., Kyushu, Japan) were used to observe the surface and cross-section structure and the morphology of the film, which can also be used to obtain roughness data.

The chemical composition and functional groups on the membrane surface were characterized using Fourier transform infrared spectroscopy (NICOLET 6700, Thermo Fisher Scientific Inc., Massachusetts, Franklin, MA, USA), X-ray photoelectron spectroscopy (Axis Ultra DLD, Shimatsu, Ltd., Kyushu, Japan) and Raman spectroscopy (inVia Reflex, Renishaw Inc., Gloucestershire, UK).

The static contact angle was measured by the seat drop method, and the wettability of the film was tested by a contact angle measuring instrument (DCAT21, Germany). One drop of 3 μL droplets was placed on the surface of the membrane and stabilized for 10 s before capturing images. The contact angle between the solid and liquid was calculated by software. A sample was measured at least five times at different positions and the average value was calculated.

### 2.7. Determination of the Performance of Forward Osmosis Membranes

FO performance testing included water flux testing, salt interception rate testing and reverse salt flux testing. The water flux of a membrane is the volume of water passing through the forward osmosis membrane per unit area per unit time. Using deionized water and 2 mol/L NaCl solution as raw materials (FS), an extraction solution (DS), an effective membrane area of 24 cm^2^, a temperature of 25 °C and a flow rate of 200 mL/min, the volume of water passing through the forward osmosis membrane per unit area per unit time was determined, denoted as the water flux of the membrane. The equation (Jw, Lm^−2^h^−1^, LMH) is shown in Equation (1). Using the relationship between conductivity and the solute concentration at a certain temperature, the concentration of salt permeated in the extraction solution was calculated by measuring the change in conductivity in the extraction solution. A 1 g/L MgSO_4_ solution and an 0.2 g/L NaF solution were used as FS, and a nonelectrolyte (glucose) was used as the DS to test the membrane retention rate. The equation (g/L) is shown in Formula (2). Using deionized water and a 2 mol/L MgSO_4_ solution as the FS and DS, the reverse salt diffusion (Js, mol m^−2^ h^−1^, abbreviated as mMH) from the raw material to the extraction side is shown in Formula (3) [40].

(1) $$J_{W}=\frac{∆V}{∆t}\frac{1}{A}$$

where ∆V, ∆t and A, respectively, represent the permeate volume of the raw material liquid (L), the membrane service time (h) and the effective membrane area (m^2^).

In addition [15]:

(2) $$R=(1\;-\;\frac{C_{p}}{C_{f}})\;\times \;100\%$$

where R, $C_{p}$ and $C_{f}$ represent the interception rate, the salt concentration of the draw solution (g/L) and the salt concentration of the feed solution (g/L), respectively.

(3) $$J_{s}=\frac{C_{t}V_{t}-C_{0}V_{0}}{∆t}\frac{1}{A}$$

where $C_{0}$ and $V_{0}$ are the salt concentration and feed rate at the beginning, and $C_{t}$ and $V_{t}$ are the salt concentration and feed rate after t h, respectively.

## 3. Results and Discussion

**Figure 1:**
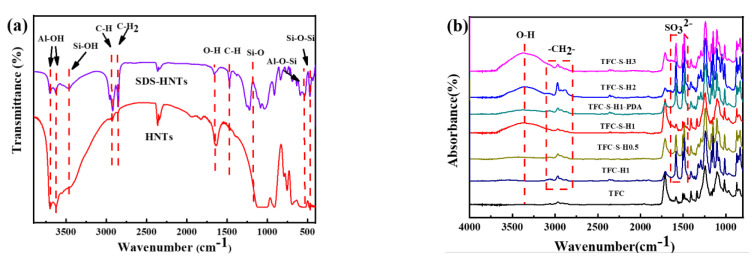

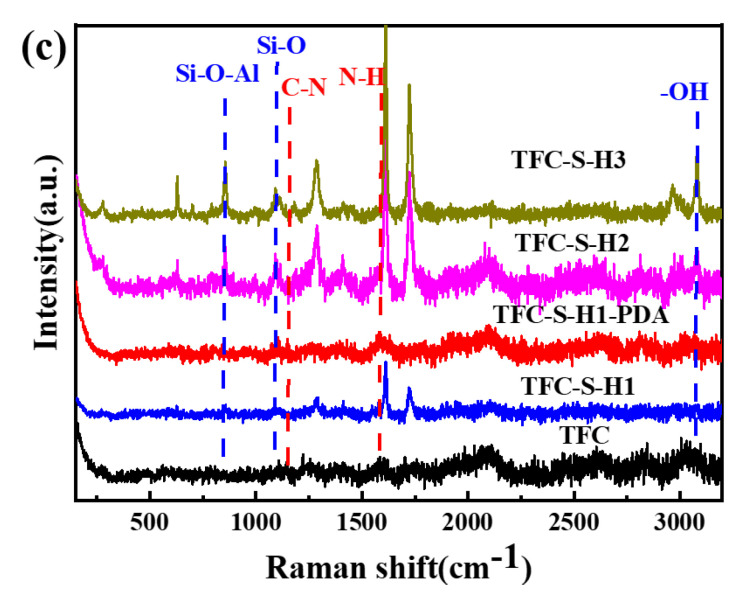
(**a**) Infrared spectra of HNTS and SDS−HNTS powder; (**b**) infrared spectra of TFC films and films with different contents of modified materials; (**c**) Raman spectra of TFC films and films with different contents of modified materials.

To verify the successful synthesis of SDS−HNTS with PDA, the modified film composition was tested by infrared spectroscopy and Raman spectroscopy, as shown in Figure 1. First, the infrared spectrum of HNTs in Figure 1a shows that the absorption peaks at 3707 cm^−1^, 3616 cm^−1^, 986 cm^−1^ and 913 cm^−1^ are the characteristic absorption peaks of HNTs. Among them, the absorption peaks at 3500–3750 cm^−1^ correspond to the stretching vibration of −OH in halloysite, 986 cm^−1^ and 913 cm^−1^ correspond to the deformation vibration of Al and the stretching vibration of Al, 540 cm^−1^ corresponds to the bending vibration of Al−O−Si and 466 cm^−1^ corresponds to the stretching vibration of Si−O−Si [41,42,43,44,45]. Furthermore, after modification with sodium dodecyl sulfate, the absorption peak at 1470 cm^−1^ is assigned as the stretching vibration of C−H and 2800 cm^−1^ and 2913 cm^−1^ are assigned as the stretching motion of C−H bonds and the asymmetric bending motion of H−C−H bonds in SDS molecules. In conclusion, the dispersion of HNTS in water is successfully modified by sodium dodecyl sulfate, which is also beneficial for the great improvement in the hydrophilicity of the membrane [7].

The infrared spectra of the forward osmosis membrane with SDS−HNTs are shown in Figure 1b. First, the results show that the absorption peaks at 1400–1680 cm^−1^ in the forward osmosis membrane can be attributed to polyamide. Among them, the peak at 1650 cm^−1^ corresponds to the vibration absorption peak of the amide I band and 1534 cm^−1^ corresponds to the vibration characteristic peak of the amide II band, which can be attributed to the tensile vibration of −CONH− and the stretching vibration of C−N [20]. Therefore, this indicates that the polyamide membrane is successfully synthesized by the polymerization reaction of homophthaloyl chloride (TMC) and m−phenylenediamine (MPD) [21]. With the addition of SDS−HNTs, the absorption peaks at 1580 cm^−1^ and 3392 cm^−1^ of SDS−HNTs appear and increase with an increase in SDS−HNTs content, confirming the successful addition of SDS−HNTs into the polyamide membrane. In addition, the infrared absorption peak of −C=O at 1470 cm^−1^ appears in the polyamide membrane, indicating that the polyamide membranes are modified by PDA.

Furthermore, the chemical composition of the polyamide membrane was analyzed by laser confocal Raman spectroscopy, as shown in Figure 1c. First, the Raman peak at 1108 cm^−1^ corresponds to the C=C stretching vibration of the polyamide. With the addition of SDS−HNTs, the characteristic peak at 860 cm^−1^ is attributed to the deformation vibration of Si−O−Al, 1144 cm^−1^ corresponds to the Si−O bond and 3082 cm^−1^ is the hydroxyl translation of halloysite nanotubes; thus, the characteristic peaks at 860 cm^−1^, 1144 cm^−1^ and 3082 cm^−1^ confirm the successful modification of the polyamide membrane by SDS−HNTs [46]. In addition, the absorption peaks at 1350 cm^−1^ and 1601 cm^−1^ correspond to C−N and N−H vibrations in PDA, suggesting the successful formation of a PDA membrane on the surface of the polyamide membrane [47].

**Figure 2:**
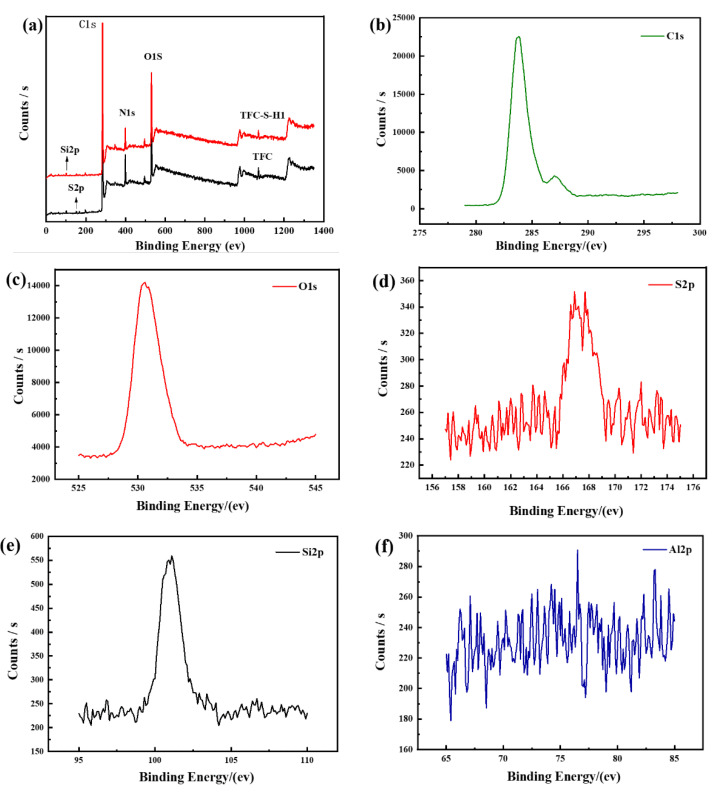
XPS diagram of the TFC film and film with 1 wt.% modified material. (**a**) Total spectrum and (**b**–**f**) sub-spectra.

The elemental composition of the polyamide membrane modified by SDS−HNTs and PDA [48] was further analyzed by XPS, as shown in Figure 2 and Table 1. Compared with the polyamide/polysulfone forward osmosis membrane, it can be found that Si and Al elements are observed in the polyamide membrane modified by SDS−HNTs and PDA, and the element concentrations of the TFC−S−H1−PDA membrane are C 71.57, O 15.93, N 9.25, Na 0.95, Si 1.78 and C 75.71, O 14.48, N 7.18, Na 0.77 and Si 1.86. In addition, the O/N ratios of the polyamide/polysulfone forward osmosis membrane and the TFC−S−H1−PDA membrane are 1.72 and 2.02, respectively. Thus, the higher O/N ratio in the TFC−S−H1−PDA membrane can be attributed to the modification of PDA on the polyamide membrane (the O/N ratio of C_8_H_11_O_2_N (DA) is 2). Thus, a dense PDA layer has been formed on the surface of the TFC−S−H1−PDA membrane [49].

**Figure 3:**
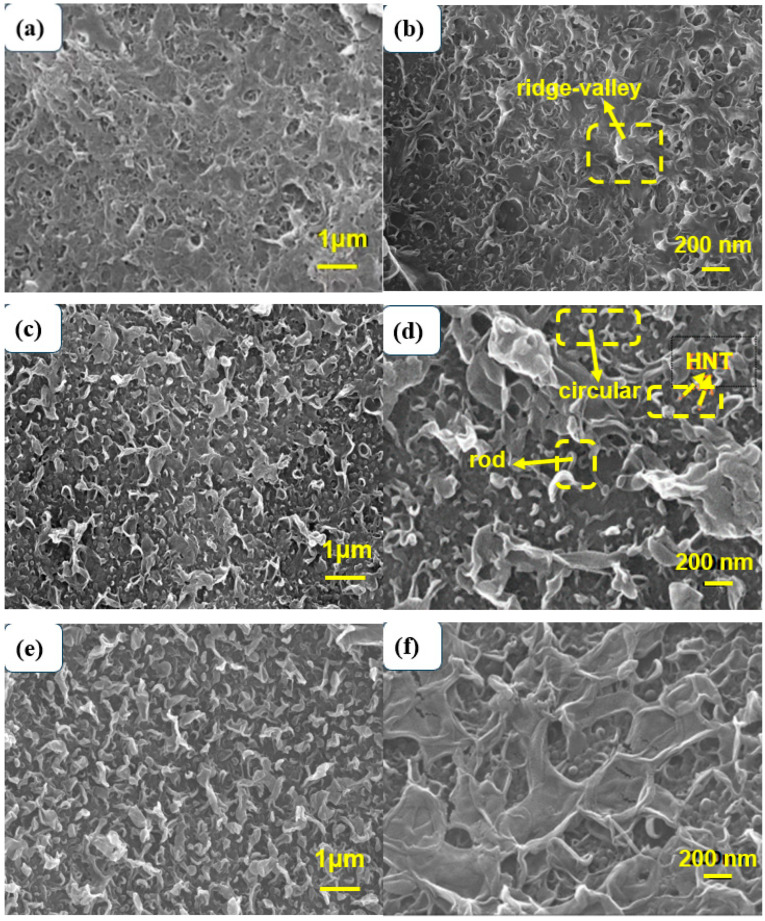

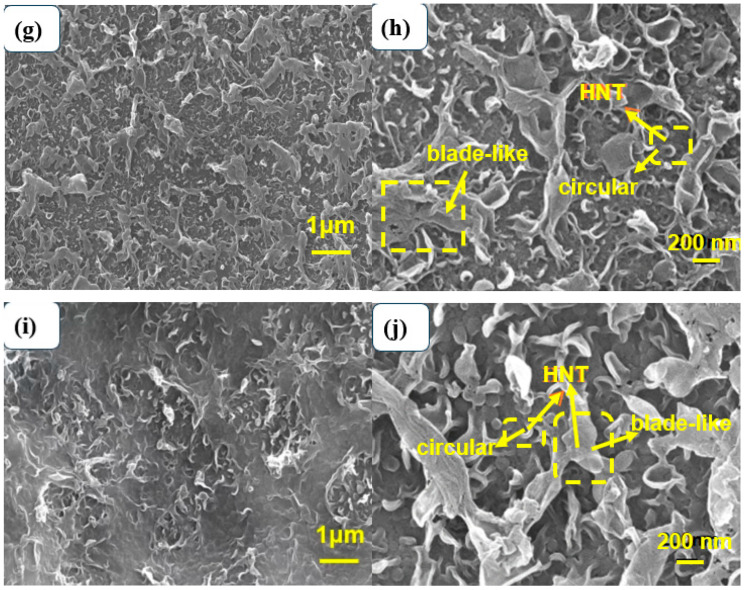
SEM images of TFC films at different magnifications and films with different contents of modified materials: (**a**,**b**) TFC, (**c**,**d**) TFC−S−H1, (**e**,**f**) TFC−S−H1−PDA, (**g**,**h**) TFC−S−H2, (**i**,**j**) TFC−S−H3.

The microstructure of the polyamide membrane modified by SDS−HNTs and PDA is shown in Figure 3. In Figure 3a,b, it can be clearly seen that the polyamide membrane exhibits a typical asymmetric “ridge−valley” structure. With the addition of SDS−HNTs, halloysite nanotubes appear on the surface of the polyamide membrane [50]. At the same time, the “ridge−valley” structure of the polyamide membrane significantly transforms into rod-, circular- and blade−like structures. This is because during the IP reaction process, the substrate surface HNTs can improve the diffusion ability of MPD in the reaction zone and promote MPD dissolution [51]. At the same time, the presence of SDS can disrupt the interface stability during the MPD diffusion process of the IP process, resulting in a rougher and looser PA layer. Therefore, the addition of SDS−HNTs promotes the formation of a highly cross-linked thick PA active layer [52], leading to the tendency of polyamide films with layered structures forming curly leaf-like structures. With 1 wt.% SDS−HNTs, a uniform and small circular structure appears on the surface of the polyamide membrane. With 3 wt.% SDS−HNTs, the blade-shaped structures of the polyamide membrane are easily expanded and form a folded structure, confirming that the curing process of the polyamide membrane is changed by the stronger supporting effect of SDS−HNTs. Thus, it can be deduced that SDS−HNTs have been successfully added into the polyamide membrane, and that the microstructure of the polyamide membrane is changed by the transition of the curing process [53]. In addition, after further modification with PDA, Figure 3e,f shows the formation of a dense PDA layer at the top of the polyamide film, indicating that the structure of the polyamide film has been successfully changed.

**Figure 4:**
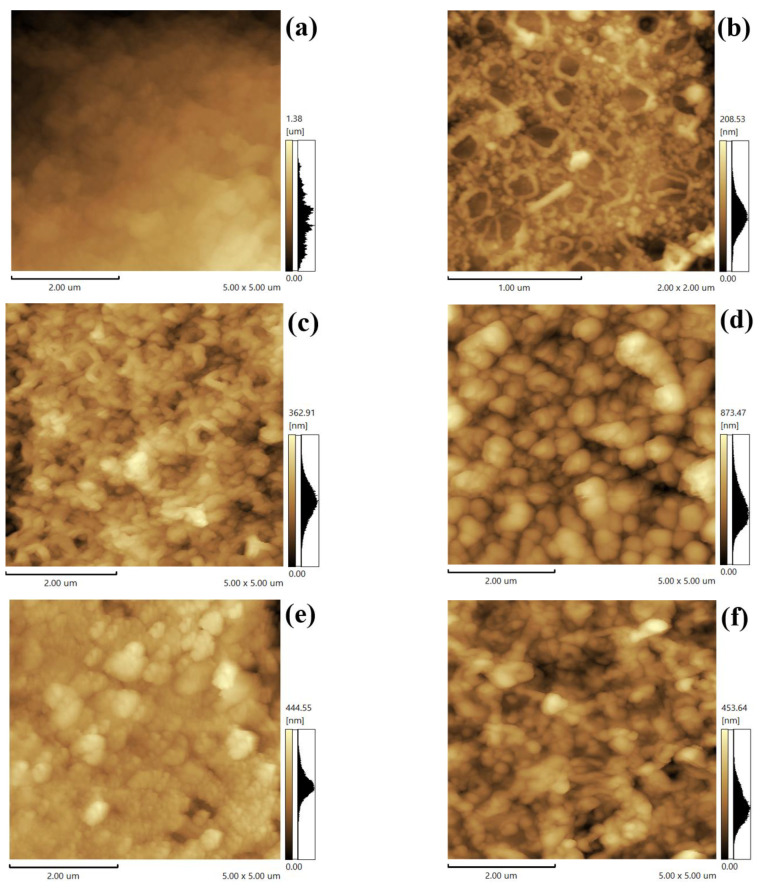
Microtopography of surface roughness: (**a**) TFC, (**b**) TFC−S−H0.5, (**c**) TFC−S−H1, (**d**) TFC−S−H1−PDA, (**e**) TFC−S−H2, (**f**) TFC−S−H3.

Moreover, the microstructure of the polyamide membrane modified by SDS−HNTs was analyzed under an atomic force microscope, as shown in Figure 4. Firstly, Figure 4a shows that the surface of the polyamide membrane is relatively flat with no obvious particles. With the addition of SDS−HNTs, the curing process of the polyamide changes, resulting in the appearance of SDS−HNTs with a nanotubular structure and an increasing number of particles. As the content of SDS−HNTs increased to 3 wt.%, more folds and micro-pores appear on the surface of the polyamide membrane, implying that the ridge−valley structure of the polyamide membrane has been destroyed by the higher content of SDS−HNTs. Furthermore, with the modification by PDA, Figure 4d shows that the introduction of PDA particles with a 100 nm size caused the polyamide to be rougher, indicating that the particle size of PDA is significantly larger than polyamide.

**Figure 5:**
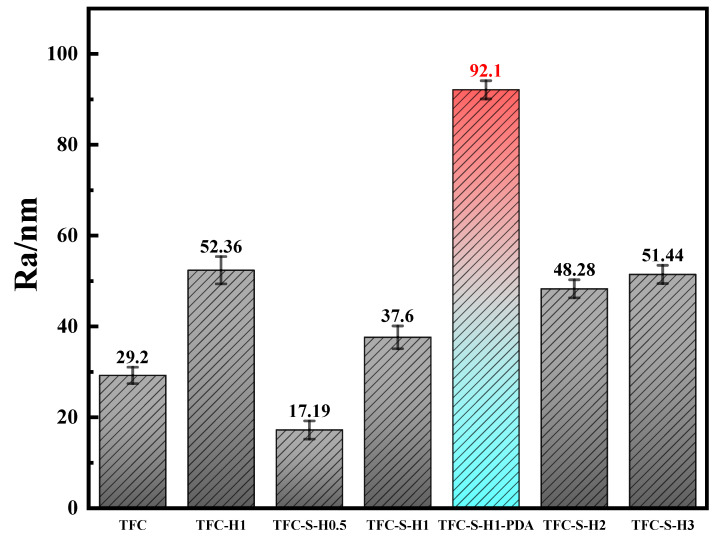
TFC membrane and Ra value with different contents of modified materials.

The surface roughness of polyamide modified by SDS−HNTs and PDA was also analyzed by atomic force microscopy, as shown in Figure 5. With the addition of HNTs, the surface roughness of the polyamide membrane clearly increases, owing to the fact that HNTs promote interfacial polymerization and improve the surface roughness of the polyamide membrane [54]. With the addition of SDS−HNTs, the surface roughness of the polyamide membrane decreases first and then increases, which may be because the surfactants not only act as the dispersants of HNTs, but also react with water during interfacial polymerization. Thus, the roughness of the polyamide membrane with addition of SDS−HNTs is higher, and thus the effective filtration area of the membrane is also larger. Meanwhile, the hydrophilic groups in SDS−HNTs can also significantly enhance the hydrophobicity of the polyamide membrane. Simultaneously, with the further addition of PDA, the surface of the polyamide membrane is covered with a layer of large particles of PDA and the surface charge of the membrane can be increased by the fact that PDA contains self-polymerized hydrophilic functional groups (phenolic hydroxyl groups) [55].

**Figure 6:**
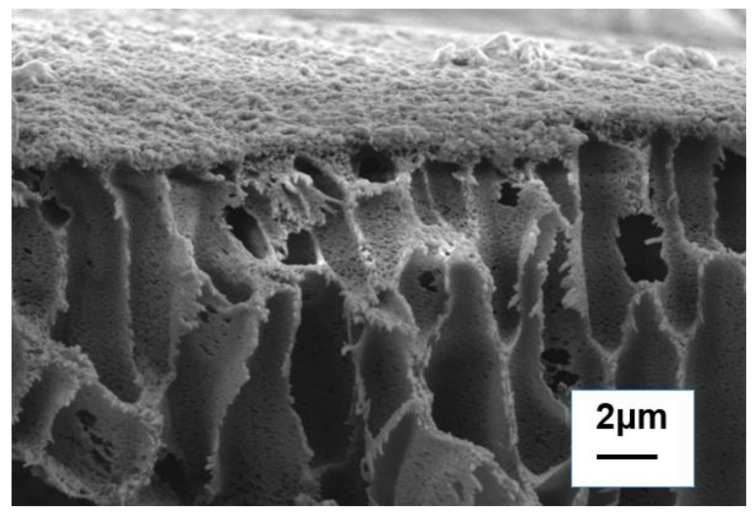
Cross-sectional morphology of the TFN−S−H1 membrane.

Figure 6 shows the cross-sectional morphology of the TFN−S−H1 membrane. Firstly, in the polysulfone layer of the forward osmosis membrane, the orderly arranged finger-like structure provides an effective water channel. Secondly, a polyamide layer can be clearly observed on the surface of the polysulfone. There are numerous convex structures on the surface of the polyamide, which may be due to the wrinkle structure induced by the addition of SDS−HNTs. Therefore, the modification of halloysite SDS−HNTs and PDA significantly improves the microstructure of the polyamide membrane, which is conducive to improving its permeability.

**Figure 7:**
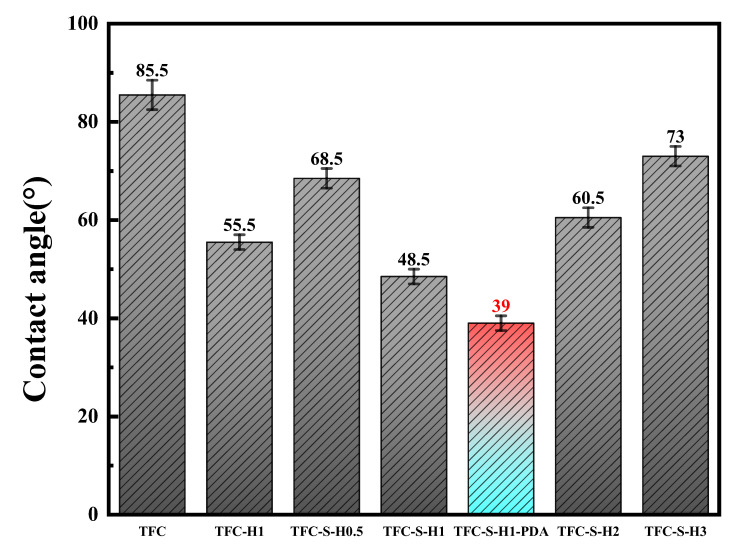
Contact angle of the TFC membrane and membranes with different contents of modified materials.

The roughness of a surface directly affects the water contact angle [53]. The water contact angles of the polyamide/polysulfone forward osmosis membrane modified by SDS−HNTs and PDA are shown in Figure 7. With the increase in SDS−HNT content, the contact angle of the polyamide membrane decreases significantly at first and then increases, owing to the clustering of a large amount of SDS−HNTs. With the PDA modification, the contact angle of the TFC−S−H1−PDA polyamide membrane further decreases to 39°, which is about 20% lower than the TFC−S−H1 polyamide membrane. As a consequence, the wettability of the polyamide membrane is improved by the addition of SDS−HNTs rich in −OH groups and PDA rich in −NH_3_ groups, and the dissolution and diffusion of water molecules on the membrane surface are promoted. The mass transfer resistance is reduced. In addition, the hydrophilic HNTs can absorb water through the capillary effect, further promoting the elevation of the water flux in the polyamide membrane [15].

**Figure 8:**
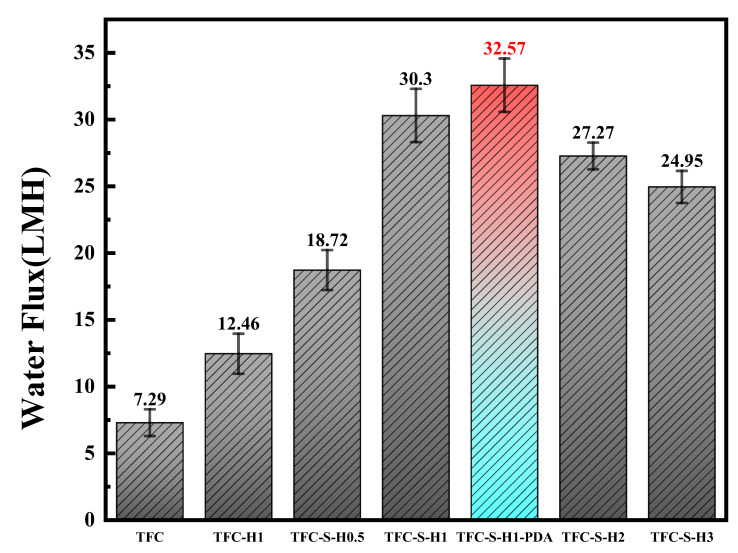
Water flux of the TFC membrane and membranes with different contents of modified materials.

The application of polyamide forward osmosis membranes is directly affected by permeability. Thus, the water fluxes of the polyamide/polysulfone forward osmosis membrane modified by SDS−HNTs and PDA are shown in Figure 8. First, the water flux of the TFC polyamide membrane is only 7.29 Lm^−2^h^−1^, restricting its application in emergency rescue. With the addition of HNTs, the water flux of the polyamide membrane increases rapidly. Furthermore, with the addition of SDS−HNTs, the water flux of the TFC−S−H1 polyamide membrane increases to 30.3 Lm^−2^h^−1^, which is approximately 4 times higher than TFC. Nevertheless, a higher amount of SDS−HNTs are aggregated in the polyamide membrane, leading to a decrease in the water flux. In addition, when the polyamide membrane modified by SDS−HNTs is further modified by PDA, the water flux of the TFC−S−H1−PDA polyamide membrane increases to 32.57 Lm^−2^h^−1^, which is 2.27 Lm^−2^h^−1^ higher than that of the TFC−S−H1 polyamide membrane. Therefore, hydrophilic groups such as the hydroxyl, carboxyl and phenolic groups of PDA not only change the interface properties of the polyamide membrane, but also form a dense PDA nanoparticle membrane, thereby increasing the surface roughness and the hydrophilicity of the polyamide/polysulfone forward osmosis membrane. Hence, the synergistic effect of SDS−HNTs and the dense PDA nanoparticle membrane significantly improves the water flux of the polyamide/polysulfone forward osmosis membrane, improving its effect in emergency rescues.

**Figure 9:**
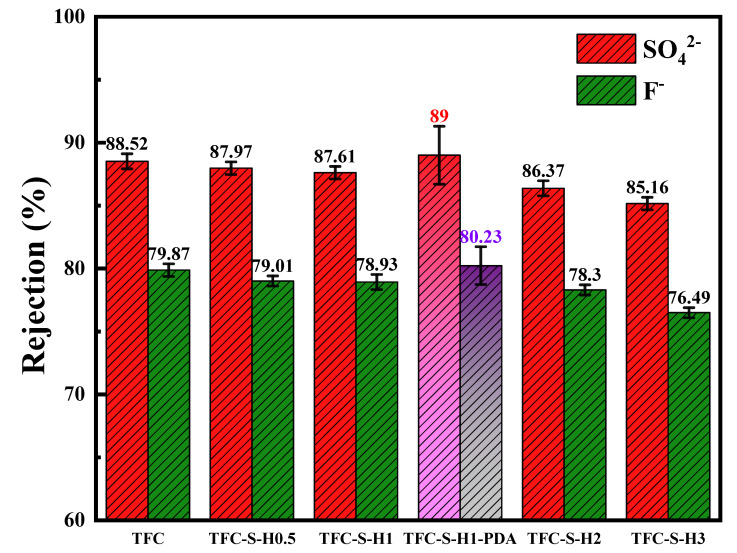
The interception rate of sulfate ions and fluoride ions of the TFC membrane and membranes with different contents of modified materials.

The rejection reflects the separation performance of the polyamide/polysulfone forward osmosis membrane. A higher rejection means a better separation effect of the membrane. Aiming at the serious problem of SO_4_^2−^ and F^−^ levels exceeding the national potable water standard, SO_4_^2−^ and F^−^ were selected to study the rejection of the polyamide/polysulfone forward osmosis membrane, as shown in Figure 9. With the addition of SDS−HNTs, the rejection of the polyamide membrane decreases, and it also maintains a high and stable rejection. This is because the SDS−HNTs increase the porosity of the membrane, and the contact of SO_4_^2−^ and F^−^ with the membrane increases at the same time. Additionally, with the further modification with PDA, it can be seen that the TFC−S−H1−PDA membrane is significantly better than the polyamide membrane solely modified by SDS−HNTs. This is due to the fact that the dense PDA nanoparticle membrane formed on the polyamide membrane achieves an effective double-layer rejection [15]. Consequently, a higher water flux and rejection of the polyamide/polysulfone forward osmosis membrane are achieved by the synergistic effect of SDS−HNTs and a dense PDA nanoparticle membrane.

**Figure 10:**
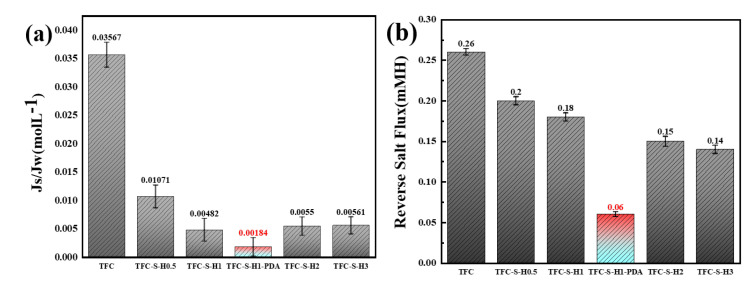
The reverse salt flux and Js/Jw of TFC−S−H modified membrane: (**a**) the reverse salt flux, (**b**) Js/Jw.

Moreover, the reverse salt flux and Js/Jw were selected to analyze the permeability of the polyamide/polysulfone forward osmosis membrane, as shown in Figure 10. With a lower reverse salt flux, the fouling tendency of the forward osmosis membrane becomes lower. The reverse salt flux of the polyamide membrane is obviously improved by the modification with SDS−HNTs and PDA. For example, the reverse salt flux of TFC−S−H1−PDA was lowered to 0.06 mMH, confirming the excellent permeability of the polyamide/polysulfone forward osmosis membrane. In addition, Js/Jw is also an important performance metric of permeability. The Js/Jw of the polyamide membrane also decreases rapidly after modification with SDS−HNTs and PDA, which is lowered to 0.0018 for TFC−S−H1−PDA. Thus, the lower the Js/Jw value, the higher the selectivity of the membrane. The highest reverse salt flux and Js/Jw indicate that TFC−S−H1−PDA modified by SDS−HNTs and PDA possesses an excellent synergistic effect [56].

**Figure 11:**
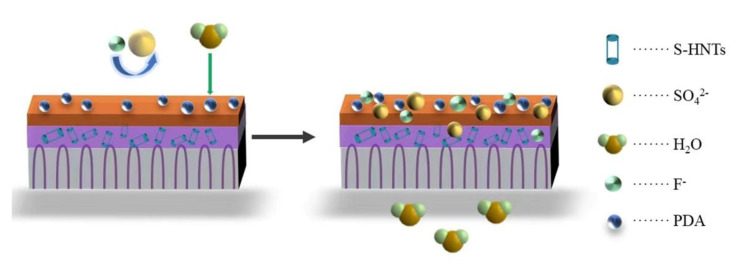
Permeation mechanism diagram of the TFC−S−H−PDA polyamide membrane.

Figure 11 shows the permeation mechanism of the polyamide/polysulfone forward osmosis membrane modified by SDS−HNTs and PDA. It can be seen that the polysulfone layer of the polyamide/polysulfone forward osmosis membrane provides regularly arranged finger-like channels, which can increase the water flux of the forward osmosis membrane. Subsequently, the polyamide layer of the polyamide/polysulfone forward osmosis membrane modified by SDS−HNTs and PDA is characterized by the staggered distribution of HNTs and a dense PDA nanoparticle membrane on the polyamide membrane, which significantly improves the permeability of the polyamide membrane. However, the high water flux leads to a decrease in the rejection rate, and then the polyamide/polysulfone forward osmosis membrane is further improved by the dense PDA nanoparticle membrane. As a result, a high-performance forward osmosis membrane with an excellent water flux, rejection rate, reverse salt flux and Js/Jw is finally obtained, providing technical support for the emergency application of forward osmosis membranes.

**Figure 12:**
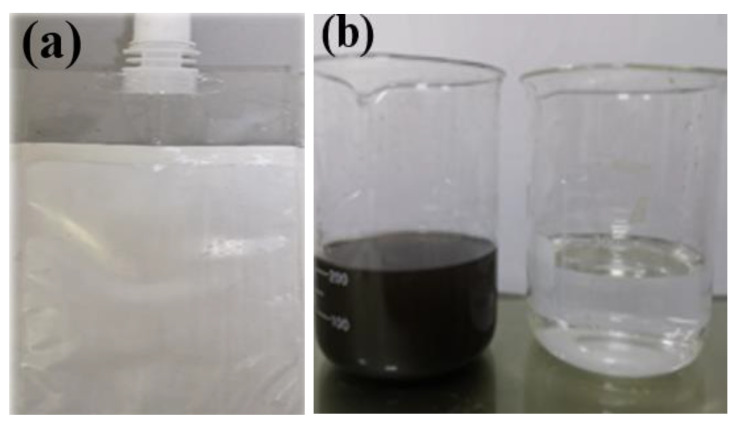
(**a**) The forward osmosis emergency water bag; (**b**) examples of emergency water bag use.

A forward osmosis emergency water bag has been successfully prepared and applied in Shendong Coal Mine Co., Ltd (Yulin, China), as shown in Figure 12a. It is expected to be distributed to other mining groups and fields, effectively to ensure that pure drinking water can be provided to trapped people in emergency situations and to extend the rescue time. The process of coal mine tunnel water filtration was simulated, and the comparison of before and after is shown in Figure 12b. In the left beaker of the figure, there is an extract composed of glucose, fructose and edible salt. On the right of the figure is the coal mine tunnel water filtered by a water bag. After filtering for a period of time, the filtered tunnel water has become pure and without obvious particles or foreign objects. In order to verify whether it meets the drinking water standards, a third-party testing agency was contacted to conduct filtered water standard testing (see Attachment 1 and Attachment 2 for the test results of Shendong mine water and filtered water, respectively).

Table 2 shows the results of tunnel water detection and the national standard safe drinking water limit after filtration with a positive osmosis water bag. It can be seen that the water quality of the mine water after being filtered by emergency water bags already meets the national standard for drinking water, demonstrating an excellent ion retention rate and providing an effective way to supply drinking water for emergency rescues in coal mines.

## 4. Conclusions

In this study, the emergency technology of filtering mine water into drinking water will effectively extend the lives of trapped people. Thus, a highly hydrophilic polyamide/polysulfone forward osmosis composite membrane was prepared by a phase conversion and interfacial bonding method. The effects of polydopamine (PDA) and modified halloysite (SDS−HNTs) on the microstructure and permeability performance of polyamide membranes were investigated, and the results are shown in the following list:

(1) The compositions of SDS−HNTs and PDA were analyzed by IR as well as Raman, which showed significant compositional differences, indicating that SDS−HNTs and PDA were successfully encapsulated in the composite film.

(2) By characterizing the microstructure of the modified film, it was found that the ridge and valley structure of the polyamide film was significantly changed to rod-, ring- and blade-like structures. Then, the surface of the polyamide film was covered by a dense layer of PDA nanoparticles, which further increased the roughness of the modified film.

(3) The polyamide membranes modified by SDS HNTs and PDA possessed both the best water flux and rejection rate, confirming the synergistic effect of the composite modification, showing excellent hydrophilicity and permeability and providing great convenience for emergency filtration of coal mine water.

Real mine water has been filtered already in previous work, which achieved good results, but the water flux was not high enough to support trapped people to obtain sufficient drinking water. Therefore, this article focuses on improving the water flux of the filtration membrane. Currently, a trial analysis is being conducted on chemical wastewater, river water and other water, and the research on applications of forward osmosis water bags will be focused on in the future.

## Figures and Tables

**Table 1:** The XPS elemental concentrations of the TFC−S−H modified membrane (atomic percentages).

Sample Name	C (%)	O (%)	N (%)	Na (%)	Si (%)	O/N
TFC	71.57	15.93	9.25	0.95	1.78	1.72
TFC−S−H1−PDA	75.71	14.48	7.18	0.77	1.86	2.02

**Table 2:** The detection value of tunnel water after filtration and the national standard safe drinking water limit.

Detection Result/mg L^−1^	Testing Items		
	*E. coli*	Sulfate	Fluoride
Filter tunnel water	ND	32	0.73
National Standard for drinking water	ND	250	1.0

ND indicates that *E. coli* is extremely low and beyond the range of the instrument.

## Data Availability

Not applicable.
